# Characteristics and Risk Factors for Delirium in Critically Ill Cardiac Surgery Patients: An Observational Study

**DOI:** 10.3390/nursrep16060184

**Published:** 2026-05-28

**Authors:** Simone Amato, Vincenza Giordano, Giuliano Anastasi, Lisa Scaramozzino, Michela Maccari, Giulia Fattore, Caterina Mercuri, Maria Catone, Francesco Gravante

**Affiliations:** 1 Cardiac Intensive Care Unit, San Camillo Forlanini Hospital, 00152 Rome, Italy; simone.amato@uniroma1.it; 2 Department of Public Health, University of Naples Federico II, 80131 Naples, Italy; vincenza.giordano@unina.it; 3 Department of Medicine and Surgery, University of Enna “Kore”, 94100 Enna, Italy; 4 Level I Emergency Department, Palidoro Unit, Bambino Gesù Children’s Hospital, IRCCS, 00165 Rome, Italy; l.scaramozzino@studenti.unitelmasapienza.it; 5 USI—Lido di Ostia, 00118 Rome, Italy; miki.maccari@gmail.com; 6 Emergency Medical Service (EMS) 118, Heart Life Croce Amica Srl., 00166 Rome, Italy; g.fattore@studenti.unitelmasapienza.it; 7 Department of Clinical and Experimental Medicine, University Magna Grecia, 88100 Catanzaro, Italy; c.mercuri@unicz.it; 8 Local Health Authority Naples 2 North, 80027 Napoli, Italy; 9 Department of Critical Care, “San Giuseppe Moscati” Hospital, Local Health Authority of Caserta, 81100 Caserta, Italy; francesco.gravante@aslcaserta.it

**Keywords:** delirium, risk factors, cardiac surgery, nursing, ICU, intensive care unit

## Abstract

**Background/Objectives**: Delirium is a frequent and clinically significant complication in cardiac surgery patients and is associated with prolonged mechanical ventilation, longer Intensive Care Unit (ICU) stay, increased mortality, and long-term cognitive impairment. However, evidence regarding perioperative factors associated with delirium occurrence in cardiac surgery ICU patients remains limited. This study aims to investigate clinical factors associated with postoperative delirium in cardiac surgery patients admitted to the ICU. **Methods**: A single-center, prospective, observational study was conducted in a 14-bed cardiothoracic ICU in central Italy. Consecutively enrolled adult patients undergoing cardiac surgery were assessed for delirium using the Italian-validated Intensive Care Delirium Screening Checklist (ICDSC) every eight hours for five days. Univariate analysis and multivariate logistic regression were performed to identify factors associated with delirium occurrence. **Results**: A total of 175 patients were included, and delirium occurred in 44.6%. In the univariate analysis, patients with delirium presented significantly longer mechanical ventilation (10.5 vs. 8.0 h; *p* = 0.04) and higher APACHE II scores (*p* = 0.01). In the multivariable analysis, lower Glasgow Coma Scale (GCS) scores were independently associated with delirium occurrence (OR = 0.84; 95% CI: 0.71–0.99; *p* = 0.04). Urgent admission (OR = 2.02; *p* = 0.06) and mean arterial pressure (OR = 0.97; *p* = 0.08) did not reach statistical significance in the multivariable model. **Conclusions**: Delirium is highly prevalent after cardiac surgery. Lower postoperative GCS scores may represent an early marker of postoperative neurological vulnerability associated with delirium occurrence. Further multicenter studies are warranted to improve delirium risk stratification and clarify the mechanisms underlying postoperative cognitive dysfunction.

## 1. Introduction

Delirium is an acute neuropsychiatric syndrome characterized by disturbances in attention, awareness, and cognition, typically exhibiting a fluctuating clinical course [1]. Despite its high clinical relevance, the underlying pathophysiological mechanisms remain incompletely understood [2]. Delirium may present in hyperactive, hypoactive, or mixed motor subtypes, each associated with distinct behavioral manifestations that can complicate timely recognition and management in intensive care unit (ICU) settings [3]. Due to its fluctuating nature, delirium is frequently under-recognized [4,5], particularly among critically ill patients, highlighting the importance of identifying populations at increased risk to support early detection and preventive strategies [5,6,7].

Delirium is among the most common neurological complications in critical care and affects a substantial proportion of ICU patients [8,9]. Patients undergoing cardiac surgery appear particularly vulnerable because of the interaction between baseline clinical frailty and perioperative stressors, including hemodynamic instability, extracorporeal circulation, prolonged mechanical ventilation, and systemic inflammatory responses [7]. Reported incidence rates of postoperative delirium after cardiac surgery are up to 50% [4,10].

In addition to perioperative factors, the ICU environment itself may contribute to delirium development through sleep disruption, continuous artificial lighting, noise exposure, physical immobilization, and social isolation [9,10,11]. The clinical impact of ICU delirium extends beyond the acute hospitalization phase and has been associated with prolonged mechanical ventilation, longer ICU and hospital lengths of stay, increased mortality, and long-term cognitive impairment [8,12].

Given these adverse outcomes, current clinical guidelines emphasize the importance of early identification of patients at higher risk for delirium. In general ICU populations, predictive tools such as the PREDELIRIC (PREdiction of DELIRium in ICu patients) model have been developed to estimate delirium risk within the first 24 h of ICU admission using multiple clinical variables [13]. Although PREDELIRIC has demonstrated acceptable performance in mixed medical–surgical ICU populations [13,14], its applicability in cardiac surgery patients remains uncertain [15,16]. In this setting, acute postoperative physiological and neurological alterations may play a more prominent role than chronic baseline characteristics, potentially limiting the performance of static prediction models [7]. Therefore, further research is needed to better characterize the clinical factors associated with delirium development in cardiac surgery patients during the immediate postoperative period.

### Aim

This study aims to evaluate the association between early postoperative clinical, neurological, and hemodynamic parameters and delirium occurrence in patients admitted to cardiothoracic ICUs after cardiac surgery. Additionally, the study sought to characterize the clinical profile of patients who developed postoperative delirium in this setting.

## 2. Materials and Methods

### 2.1. Study Design and Ethical Considerations

A single-center, prospective, observational study was conducted in a cardiothoracic ICU. The study design and reporting adhered to the Strengthening the Reporting of Observational Studies in Epidemiology (STROBE) guidelines. The protocol was approved by the Lazio 4 Ethics Committee (Ref: 91-2024), and written informed consent was obtained from all participants before enrollment.

### 2.2. Setting and Participants

Data collection was conducted between October 2024 and May 2025 at a university hospital in Central Italy (1000-bed capacity). The study was performed in a 14-bed postoperative cardiac surgery ICU with a nurse-to-patient ratio of 1:2. Consecutive patients were enrolled according to the following inclusion criteria: (a) age ≥ 18 years, (b) underwent cardiac surgery, (c) able to understand Italian, and (d) ICU length of stay (LOS) ≥ 24 h. Patients were excluded if they (a) had a diagnosis of delirium at ICU admission; (b) were in a profound coma unrelated to sedation (Richmond Agitation–Sedation Scale [RASS] score of −4 or −5); (c) had a pre-existing cognitive impairment or major psychiatric or neurological disorders; or (d) were transferred from another ICU, where pre-transfer exposures could not be reliably assessed.

### 2.3. Variables

Sociodemographic variables included age, sex, educational level, ethnicity, smoking status, and alcohol consumption. Clinical variables included type of surgery, use of mechanical circulatory support, continuous renal replacement therapy (CRRT), mean arterial pressure (MAP), duration of extracorporeal circulation, duration of mechanical ventilation, ICU LOS, sedation requirements, metabolic acidosis, urgent admission, infection, and admission category.

### 2.4. Delirium Assessment and Measures

Delirium was assessed using the Italian version of the Intensive Care Delirium Screening Checklist (ICDSC) [17]. The ICDSC is an eight-item screening instrument evaluating (1) altered level of consciousness, (2) inattention, (3) disorientation, (4) hallucinations and psychosis, (5) agitation or motor retardation, (6) altered speech or mood, (7) sleep–wake cycle disturbances, and (8) fluctuation of symptoms. Each item was scored dichotomously as present (1) or absent (0), yielding a total score ranging from 0 to 8. For the altered level of consciousness item, scores were assigned according to the following criteria: no response or response only to vigorous stimulation (0 points); response to mild or moderate stimulation or the presence of drowsiness, indicating an altered level of consciousness (1 point); normal wakefulness or easily arousable sleep (0 points); and hypervigilance or exaggerated responsiveness to normal stimulation (1 point). All remaining items were scored as either present (1) or absent (0). Based on established cut-off values, an ICDSC score of ≥4 indicated clinical delirium, whereas scores between 1 and 3 indicated subsyndromal delirium. The ICDSC has demonstrated high sensitivity (99%) and moderate specificity (64%) for delirium detection and has been validated and adapted for use in the Italian population [2,18].

Baseline delirium risk was estimated within the first 24 h of ICU admission using the PREDELIRIC model [2]. Disease severity and mortality risk were assessed using the Acute Physiology and Chronic Health Evaluation II (APACHE II) score [19], which incorporates 12 physiological parameters, age, comorbidities, and the Glasgow Coma Scale (GCS).

### 2.5. Outcome

The primary outcome was the incidence of delirium during an ICU stay. Secondary outcomes included survival, duration of mechanical ventilation, and ICU LOS.

### 2.6. Data Collection

All consecutive critically ill patients included in the study underwent a two-stage assessment process. First, baseline delirium risk was estimated using the PREDELIRIC score within the first 24 h of ICU admission. Subsequently, delirium screening was performed using the ICDSC after recovery from sedation and achievement of an adequate level of consciousness.

To establish a neurologically reliable baseline and minimize conceptual overlap with delirium assessment, the GCS was assessed after emergence from anesthesia and discontinuation or substantial reduction in sedative medications potentially affecting neurological evaluation. Postoperative sedation was standardized using short-acting agents, primarily propofol and remifentanil, according to institutional weaning protocols.

Delirium screening was initiated only after extubation and once patients achieved an adequate arousal level (RASS ≥ −3). ICDSC assessments were performed every 8 h for 5 days by a critical care nurse specifically trained in ICDSC administration.

After completion of the assessments, all study variables were recorded in a standardized data collection form. An alphanumeric identification code was assigned to each participant to ensure confidentiality and anonymization of the dataset.

### 2.7. Sample Size

The minimum sample size was calculated assuming an expected delirium prevalence of 32% [20], with an absolute precision of 0.07 and a 95% confidence interval. Based on these assumptions, a minimum sample size of 171 critically ill patients was required.

### 2.8. Data Analysis and Statistical Methods

Statistical analyses were performed using SPSS version 22.0 (IBM Corp., Armonk, NY, USA). Continuous variables are presented as mean and standard deviation (SD) or median and interquartile range (IQR), according to data distribution. Categorical variables are reported as frequencies and percentages.

Comparisons between patients with and without delirium were performed using the independent samples *t*-test, Mann–Whitney U test, Pearson chi-square test, or Fisher’s exact test, as appropriate. A two-tailed *p*-value < 0.05 was considered statistically significant.

Variables associated with delirium at *p* < 0.10 in univariate analyses were initially considered for inclusion in the multivariable logistic regression model. To reduce the risk of overfitting and multicollinearity, clinically overlapping variables were carefully evaluated before model construction. Specifically, the APACHE II score was excluded from the final multivariable model because of its conceptual and mathematical overlap with GCS, which is incorporated into the APACHE II calculation. Similarly, the duration of mechanical ventilation was excluded because it was considered a post-exposure clinical outcome rather than an early baseline predictor measurable upon ICU admission. The final multivariable regression model, therefore, included three non-collinear variables: urgent admission, MAP, and GCS. Odds ratios (ORs) and 95% confidence intervals (95% CIs) were calculated to estimate the association between each variable and delirium occurrence.

Model calibration was assessed using the Hosmer–Lemeshow goodness-of-fit test, while overall model significance was evaluated using the Omnibus chi-square test. The proportion of explained variance was estimated using Nagelkerke’s R^2^.

## 3. Results

### 3.1. Participant Flow and Delirium Characteristics

**Figure 1:**
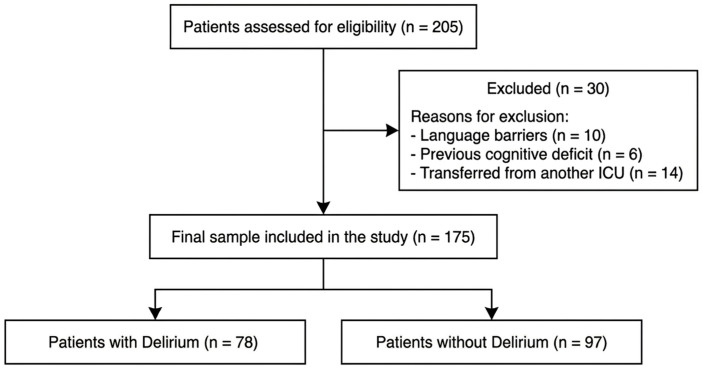
Flow diagram of patient screening and enrollment.

A total of 205 patients admitted to the ICU were screened for eligibility. Of these, 175 patients met the inclusion criteria and were included in the final analysis, while 30 were excluded. The participant selection process is summarized in Figure 1.

The cohort had a mean age of 65.48 years (SD = 9.34), and most participants were male (n = 133; 76.0%). The majority had completed high school education (n = 152; 86.9%) and were Caucasian (n = 173; 98.9%). Regarding lifestyle habits, 74.9% (n = 131) reported a history of smoking (including former and current smokers), whereas 24.6% (n = 43) reported alcohol consumption. All patients received postoperative sedation during the immediate ICU stay. Overall ICU survival rate was 95.4% (n = 167), with an ICU mortality rate of 4.6% (n = 8). Baseline demographic and clinical characteristics of the study population are presented in Table 1.

### 3.2. Comparison of Clinical Variables Between Patients with and Without Delirium

Postoperative delirium occurred in 78 patients (44.6%), whereas 97 patients (55.4%) did not develop delirium during their ICU stay. Detailed comparisons between groups are reported in Table 2.

No statistically significant differences were observed between groups regarding age (*p* = 0.16), sex (*p* = 0.79), educational level (*p* = 0.62), smoking history (*p* = 0.62), or alcohol consumption (*p* = 0.51). Similarly, no significant differences were found for infection (*p* = 0.76), metabolic acidosis (*p* = 0.75), intraoperative mechanical circulatory support (*p* = 0.76), CRRT requirement (*p* = 0.16), type of cardiac surgery (*p* = 0.36), or duration of extracorporeal circulation (*p* = 0.42).

Patients who developed delirium more frequently underwent urgent admission procedures compared with those without delirium (33.3% vs. 21.6%; *p* = 0.05). In addition, the delirium group showed a longer duration of mechanical ventilation (median: 10.5 [IQR: 6.0–19.2] h vs. 8.0 [IQR: 5.0–13.5] h; *p* = 0.04). Regarding physiological parameters at admission, patients with delirium had significantly lower mean arterial pressure (72.99 ± 10.83 mmHg vs. 76.29 ± 11.97 mmHg; *p* = 0.05) and lower GCS scores (13.95 ± 3.20 vs. 14.67 ± 1.40; *p* = 0.05). Furthermore, APACHE II scores were significantly higher among patients who developed delirium (*p* = 0.01). No statistically significant difference was observed in PREDELIRIC scores between groups (*p* = 0.39).

### 3.3. Univariate and Multivariate Analysis Results

The results of the multivariable logistic regression analysis are presented in Table 3. After adjustment for potential confounders, lower GCS scores were significantly associated with delirium occurrence. Specifically, each one-point increase in GCS was associated with a 16% reduction in the odds of delirium development (OR = 0.84; 95% CI: 0.71–0.99; *p* = 0.04). Urgent admission (OR = 2.02; 95% CI: 0.98–4.15; *p* = 0.06) and mean arterial pressure (OR = 0.97; 95% CI: 0.95–1.00; *p* = 0.07) did not reach statistical significance in the adjusted model.

The overall logistic regression model was statistically significant, as indicated by the Omnibus Test of Model Coefficients (*X*^2^ = 11.73, df = 3, *p* = 0.008). The Hosmer–Lemeshow test was non-significant (*X*^2^ = 11.08, df = 8, *p* = 0.197), indicating adequate goodness-of-fit and indicating that the predicted probabilities align well with the observed outcomes. The final model explained approximately 8.7% of the variance in delirium occurrence (Nagelkerke *R*^2^ = 0.087).

## 4. Discussion

The present study investigated clinical factors associated with delirium occurrence in a cohort of cardiothoracic ICU patients following cardiac surgery. Delirium occurred in nearly half of the study population, confirming its high prevalence in this clinical setting. In the adjusted analysis, lower GCS scores upon ICU admission were associated with delirium occurrence, whereas higher APACHE II scores and prolonged mechanical ventilation were observed among patients who developed delirium in univariate analyses.

The prevalence of delirium observed in our cohort (44.6%) is consistent with previous studies reporting incidence rates ranging between 34% [10] and 54.9% [4] following cardiac surgery. These findings further support the view that delirium remains one of the most common neurological complications in postoperative critical care. Previous evidence has also shown that postoperative delirium after cardiac surgery is associated with persistent cognitive impairment after ICU discharge [8].

In multivariable analysis, lower GCS scores were independently associated with delirium occurrence. In general ICU populations, lower GCS scores are often a direct marker of encephalopathy or severe neurological dysfunction [2]. However, the interpretation of this finding in patients undergoing cardiac surgery requires caution. Unlike general ICU populations, cardiothoracic surgical patients are commonly managed using fast-track anesthesia and early extubation protocols aimed at rapid postoperative recovery [21]. In the specific context of cardiac surgery, lower postoperative GCS scores may reflect delayed neurological recovery following extracorporeal circulation, hemodynamic instability, or residual vulnerability to postoperative cognitive dysfunction [22]. Accordingly, early postoperative neurological responsiveness may represent a more critical determinant of delirium than baseline patient complexity alone [23,24].

Patients who developed delirium also presented higher APACHE II scores in univariate analysis, consistent with previous studies linking greater illness severity to delirium occurrence in critically ill patients [2]. These findings further support the hypothesis that systemic physiological derangement contributes to cognitive dysfunction in critically ill populations [8]. Cardiothoracic surgical patients frequently present with multiple comorbidities, including hypertension, diabetes mellitus, and vascular disease, which may reduce physiological reserve and increase susceptibility to postoperative neurological complications [25]. In addition, greater illness severity may reflect systemic inflammation and impaired organ perfusion, which have been associated with delirium pathophysiology [26]. However, APACHE II did not retain statistical significance in the multivariable model. This finding is likely related to the conceptual overlap between APACHE II and GCS, since neurological responsiveness is incorporated into the APACHE II calculation. Consequently, the independent contribution of overall illness severity and postoperative neurological status could not be fully disentangled within the multivariable analysis.

The significant association between mechanical ventilation duration and delirium is also consistent with previous literature. In ICU settings, mechanical ventilation is a complex intervention that interacts dynamically with the patient’s neurological state [27]. This association suggests a reciprocal mechanism: while delirium can result from prolonged intubation due to sedation and immobilization, it also acts as a primary disruptor of respiratory weaning. A crucial pathophysiological link is the central control of ventilation [28]; as a manifestation of acute brain failure, delirium often leads to altered dyspnea perception and a disorganized respiratory drive [29]. This disorganization results in patient-ventilator asynchrony, which often necessitates higher sedative doses—further exacerbating cognitive impairment [30]. Moreover, delirium may affect the homeostatic control of carbon dioxide (CO_2_). Delirious patients often exhibit irregular breathing patterns that lead to fluctuations in PaCO_2_, which in turn influence cerebral blood flow via vasodilation or vasoconstriction [31]. Such fluctuations can aggravate cerebral ischemia or edema, creating a feedback loop that sustains the delirious state.

Finally, the PREDELIRIC score did not significantly differ between patients with and without delirium. This finding may suggest that general ICU prediction models incompletely capture the acute postoperative physiological and neurological alterations characterizing cardiac surgery patients. Conversely, early postoperative physiological indicators, particularly neurological responsiveness upon ICU admission, may better reflect the immediate postoperative vulnerability associated with delirium occurrence in this population.

### 4.1. Implications for Clinical Practice

The high prevalence of delirium observed in this study highlights the importance of systematic delirium surveillance in cardiothoracic ICU settings. The associations observed between delirium occurrence, early neurological responsiveness, illness severity, and prolonged mechanical ventilation support the need for early identification of patients with greater postoperative vulnerability. In this context, routine delirium screening with validated instruments such as the ICDSC should be integrated into standard postoperative critical care practice to facilitate timely recognition and early implementation of preventive interventions. Attention should be directed toward patients presenting with lower postoperative neurological responsiveness, as this factor may identify individuals at increased risk for delirium during the immediate postoperative phase.

Critical care nurses play a central role in delirium prevention, monitoring, and management. Key nursing interventions include optimization of sedation strategies, promotion of early mobilization, sleep preservation, cognitive reorientation, and family engagement. Furthermore, delirium management should not be limited to the acute ICU phase, as it has been associated with long-term cognitive impairment, functional decline, and reduced quality of life after discharge [32].

### 4.2. Limitations and Future Research

This study has several limitations that should be acknowledged. First, the single-center design may limit the generalizability of the findings to different healthcare settings. Second, although the study achieved the planned sample size, the relative homogeneity of the cardiac surgery cohort might have reduced variability in clinical variables and limited the identification of additional factors associated with delirium occurrence.

Furthermore, some perioperative variables, including anesthetic exposure, cumulative sedative dosage, vasoactive therapies, postoperative pain, and frailty-related characteristics, were not assessed. Consequently, some relevant contributors to postoperative delirium may not have been captured within the present analysis.

Delirium assessment was limited to the first five postoperative days; therefore, cases of late-onset delirium may have been underestimated. In addition, the observational design precludes causal inferences, and the absence of long-term follow-up limits conclusions regarding the cognitive, functional, and clinical trajectories associated with postoperative delirium and subsyndromal delirium.

Although GCS assessment was standardized following emergence from anesthesia and sedation reduction protocols, residual sedative effects may still have influenced early neurological responsiveness in some patients. Moreover, because altered consciousness represents one component of delirium assessment, some conceptual overlap between GCS and delirium-related neurological dysfunction cannot be completely excluded.

Another limitation relates to the modest explanatory power of the final multivariable model (Nagelkerke R^2^ = 0.087), suggesting that postoperative delirium following cardiac surgery is likely influenced by multiple perioperative, environmental, and patient-related factors not fully captured in the present study.

Future research should employ multicenter designs with larger and heterogeneous samples to further investigate perioperative factors associated with delirium occurrence after cardiac surgery. Additional studies incorporating longitudinal follow-up, perioperative pharmacological variables, frailty measures, biomarkers, and standardized neurological assessments may help improve risk stratification and clarify the relationship between early postoperative neurological dysfunction and delirium development. Furthermore, greater attention should be directed toward understanding the progression from subsyndromal to overt delirium and its long-term cognitive and functional consequences.

## 5. Conclusions

Delirium remains a frequent and clinically significant complication among patients admitted to the ICU following cardiac surgery, affecting nearly half of the study population in the present cohort. Patients who developed delirium presented greater overall physiological severity and longer duration of mechanical ventilation, while lower postoperative GCS scores were independently associated with delirium occurrence.

These findings suggest that early postoperative neurological responsiveness may represent a clinically relevant marker of acute postoperative vulnerability in cardiac surgery patients. However, the association between lower GCS scores and delirium should be interpreted cautiously, considering the complex multifactorial nature of postoperative delirium, the potential influence of residual sedative effects, and the possible conceptual overlap between neurological responsiveness and delirium-related alterations in consciousness.

The absence of significant differences in PREDELIRIC scores between groups may indicate that general ICU prediction models incompletely capture the acute physiological and neurological changes characterizing the postoperative cardiac surgery population. At the same time, the modest explanatory power of the final multivariable model highlights that delirium development is likely influenced by multiple perioperative, environmental, pharmacological, and patient-related factors not fully assessed in the present study.

Overall, these findings reinforce the importance of systematic delirium surveillance and close neurological monitoring during the immediate postoperative phase following cardiac surgery. Early identification of patients with greater physiological and neurological vulnerability may facilitate the timely implementation of individualized sedation management and non-pharmacological preventive strategies aimed at reducing the clinical burden of delirium in cardiothoracic ICU settings.

Further prospective multicenter studies incorporating longitudinal follow-up, perioperative pharmacological variables, biomarkers, frailty measures, and standardized neurological assessments are warranted to improve delirium risk stratification and better characterize the mechanisms underlying postoperative cognitive dysfunction after cardiac surgery.

## Figures and Tables

**Table 1:** Baseline characteristics of the study population (n = 175).

Variable	n	%
Demographic Characteristics		
Gender (male)	133	76.0%
Caucasian	173	98.9%
Education Level		
Elementary school	20	11.4%
High school	152	86.9%
Graduate	3	1.7%
Lifestyle & Habits		
Smoking habit (yes)	131	74.9%
Alcohol habit (yes)	43	24.6%
Clinical History & Admission		
Urgent admission	47	26.9%
Admission category (surgery)	172	98.3%
Survival	167	95.4%
Type of cardiac surgery		
CABG	83	47.4%
Aortic dissection	7	4.0%
Aortic valve replacement	31	17.7%
Transcatheter aortic valve implantation	3	1.7%
Heart transplant	1	0.6%
Mitral valve repair (mitral valvuloplasty)	20	11.4%
Mitral valve replacement	5	2.9%
Other	25	14.3%
Intraoperative & Clinical Factors		
Mechanical assistance during surgery (yes)	10	5.7%
CRRT (yes)	6	3.4%
Metabolic acidosis (yes)	8	4.6%
Infection (yes)	10	5.7%
	Means	SD
Age (years)	65.48	9.34
APACHE II score	12.04	5.07
Glasgow Coma Scale	14.27	2.62
Duration of extracorporeal circulation (min)	87.90	31.10
Mean Arterial Pressure (mmHg)	74.82	11.56
	Median	IQR
Duration of mechanical ventilation (h)	9	5–18
ICU length of stay (days)	4	2–5
PREDELIRIC	26.0	12.0–81.0

Legend: ICU = intensive care unit; SD = standard deviation; APACHE = Acute Physiologic Assessment and Chronic Health Evaluation II; IQR = interquartile range; CABG = coronary artery bypass graft; CRRT = continuous renal replacement therapy.

**Table 2:** Comparison of baseline and clinical characteristics between the delirium and non-delirium groups.

Variable	Non-Delirium Group (n = 97)	Delirium Group (n = 78)	*p*-Value	Effect Size
Categorical Variables, n (%)				(Cramer’s V)
Gender (male), n (%)	73 (75.3%)	60 (76.9%)	0.79 ^a^	0.02
Education (High School), n (%)	82 (84.5%)	70 (89.7%)	0.62 ^a^	0.04
Smoking Habit (Yes), n (%)	74 (76.3%)	57 (73.1%)	0.62 ^a^	0.03
Alcohol Habit (Yes), n (%)	22 (22.7%)	21 (26.9%)	0.51 ^a^	0.05
Infection (Yes), n (%)	6 (6.2%)	4 (5.1%)	0.76 ^b^	0.02
Urgent Admission (Yes), n (%)	21 (21.6%)	26 (33.3%)	0.05 ^a^	0.13
Metabolic Acidosis (Yes), n (%)	3 (3.1%)	5 (6.4%)	0.75 ^b^	0.07
Mechanical Assistance (Yes), n (%)	5 (5.2%)	5 (6.4%)	0.76 ^b^	0.02
CRRT (Yes), n (%)	2 (2.1%)	4 (5.1%)	0.16 ^b^	0.08
Type of Surgery (CABG), n (%)	48 (49.5%)	35 (44.9%)	0.36 ^a^	0.05
Continuous Variables	Mean ± SD	Mean ± SD		(Cohen’s d)
Age (years)	66.37 ± 9.27	64.37 ± 9.38	0.16 ^c^	0.21
APACHE II score	11.79 ± 5.99	12.35 ± 3.62	0.01 ^d^*	0.11
Glasgow Coma Scale	14.67 ± 1.40	13.95 ± 3.20	0.05 ^c^	0.29
Mean Arterial Pressure (mmHg)	76.29 ± 11.97	72.99 ± 10.83	0.05 ^c^	0.29
Extracorporeal Circulation (min)	86.20 ± 30.93	90.02 ± 31.38	0.42 ^c^	0.12
Non-Parametric Variable	Median [IQR]	Median [IQR]		(r)
Duration of Ventilation (h)	8.0 [5.0–13.5]	10.5 [6.0–19.2]	0.04 ^d^	0.19
ICU Length of Stay (days)	3.0 [2.0–5.0]	4.0 [3.0–5.0]	0.10 ^d^	0.15
PREDELIRIC	26.0 [22.0–31.0]	26.0 [24.0–31.0]	0.39 ^d^**	0.06

Legend: ^a^ Pearson’s chi-square test; ^b^ Fisher’s exact test; ^c^ independent samples *t*-test; ^d^ Mann–Whitney U test; * mean rank (79.4 vs. 98.6); ** mean rank (85.1 vs. 91.6). Effect size indices: Cramer’s V for categorical variables, Cohen’s d for parametric continuous variables, and rank-biserial r for non-parametric variables.

**Table 3:** Multivariable logistic regression analysis of risk factors associated with delirium.

Variables	*B*	S.E.	OR	95% CI	*p*-Value
Urgent Admission	0.701	0.368	2.02	0.98–4.15	0.06
Mean Arterial Pressure	−0.026	0.014	0.97	0.95–1.00	0.07
Glasgow Coma Scale	−0.173	0.084	0.84	0.71–0.99	0.04
**Items**	**Statistics**			***p*-Value**	
Omnibus Chi-Square	11.73			0.008	
Hosmer–Lemeshow test	11.08			0.197	

Legend: OR = odds ratio, CI = confidence interval, *B* = coefficient; S.E. = standard error.

## Data Availability

The collected data files are available upon request from the corresponding author. The data are not publicly available due to privacy and ethical restrictions.

## Public Involvement Statement

No public involvement in any aspect of this research.

## Guidelines and Standards Statement

This manuscript was drafted in accordance with the Strengthening the Reporting of Observational Studies in Epidemiology (STROBE) statement for observational studies.

## Abbreviations

The following abbreviations are used in this manuscript:

PREDELIRIC	Prediction of delirium in ICU patients
APACHE II	Acute Physiology and Chronic Health Evaluation II
ICU	Intensive care unit
CABG	Coronary artery bypass graft surgery
CRRT	Continuous renal replacement therapy
ICDSC	Intensive Care Delirium Screening Checklist
GCS	Glasgow Coma Scale
CI	Confident interval
OR	Odds ratio
STROBE	Reporting of Observational Studies in Epidemiology
IQR	Interquartile range
